# Unravelling mechanisms underlying the action principles of a community-based health promotion programme: a realist evaluation

**DOI:** 10.1186/s13690-023-01027-0

**Published:** 2023-01-19

**Authors:** Marja A. J. G. de Jong, Gerda Wink, Maria A. Koelen, Annemarie Wagemakers

**Affiliations:** 1 GGD IJsselland (Municipal Health Service), Zwolle, the Netherlands; 2 Wageningen University and Research, chair Health and Society, Wageningen, the Netherlands; 3 Department of Primary and Community Care, Radboud University Medical Centre, AMPHI Academic Collaborative Centre, Nijmegen, the Netherlands

**Keywords:** Critical realist perspective, Community-based health promotion, Action principles, Participatory action research

## Abstract

**Background:**

Since 1986, WHO has advised that applying action principles such as citizen participation and intersectoral collaboration leads to better health. However, less is known about the workability of these principles and how they trigger specific outcomes in interaction with the context. A critical realist perspective was applied to get a better understanding of what worked, and why it worked, in the context of a Dutch community-based health promotion programme (CBHPP). The aim of the study was to unravel the mechanisms underlying the action principles and find combinations of contextual factors and mechanisms that trigger outcomes in a CBHPP.

**Methods:**

In this single case study, a critical realist methodology was followed. Qualitative data used in this study originated from multiple sources and methods to ensure validity. They include evaluation sessions with coalition members (*n* = 6) and individual interviews (*n* = 6); group sessions with community workers (*n* = 1), a health broker (*n* = 1), and citizens (*n* = 12); and seven semi-annual progress reports and minutes of the coalition meetings. The collected data were then compared with the programme theory through a heuristic process of constructing, exploring, and refining context-mechanism-outcome configurations.

**Results:**

The programme initiated a variety of new activities that differed in content, intensity, duration, and number of participants, organised and implemented together with citizens. The most prominent mechanism underlying both action principles were programme-related, namely, patience, personal contact, contribution of budget, and the programme coordinator’s leadership. Another important mechanism was creating visibility, which resulted in the involvement of the municipality and a budget to sustain the programme.

**Conclusion:**

In this case study, personal contact, patience, perseverance, participatory action research activities, and visibility were found to be the most notable mechanisms underlying the citizen participation and intersectoral collaboration action principles. As the principle-based approach added value to the existing context and introduced most of the mechanisms that triggered the outcomes, it is recommended to include citizen participation and intersectoral collaboration not only as action principles but explicitly as targets in a CBHPP.

**Supplementary Information:**

The online version contains supplementary material available at 10.1186/s13690-023-01027-0.

## Background

People with a low socioeconomic status (SES) live – on average – six years less compared to those with a high SES, and the difference in healthy life expectancy between these groups is huge, e.g., almost 19 years in the Netherlands [1, 2]. Although the healthy life expectancy of people with a low SES has increased considerably in the last decade, the difference in life expectancy between the two groups has remained the same, or even become worse as a result of the Covid pandemic [3–5]. Health inequalities are a complex problem caused by the interplay between individuals, groups, communities, and multiple factors in the social, physical, and economic environment [6, 7]. There have been many studies on the causes of health inequalities [8–10]. The persistence of health inequalities within societies indicates the importance of research on the social determinants of health [11] and on policies and interventions that aim to reduce inequalities [8, 12].

Community-based health promotion programmes (CBHPPs), based on an ecological perspective, are seen as a promising approach to diminish health inequalities, as they address the social determinants of health at multiple levels as well as the interaction between these determinants and factors that impact the determinants [13–16]. In CBHPPs, citizen participation and intersectoral collaboration are essential elements, also called action principles [17–19]. These action principles contribute to health through multiple pathways and serve multiple purposes, such as programme effectiveness, the creation of supportive environments for health, and the empowerment of all stakeholders, both professionals and citizens [20]. Action principles can be defined as actions, processes, or mechanisms that help establish the impacts of a health promotion programme [21, 22]. Through citizen or community participation, described as the active involvement of citizens, or members of the priority population, in the articulation of the problem and in the development, implementation, and evaluation of health-promoting interventions [20], the context of people’s lives can be taken into account. This offers opportunities to address the social determinants of health, for example by addressing informal networks and cultural aspects. Because of their connection with the existing local situation, health promotion programmes can be more effective [23, 24]. In addition, intersectoral collaboration between professionals in health, care, and other societal sectors is regarded as crucial for working on diminishing the health gap [25, 26]. Professionals from different sectors that collaborate can achieve more than one sector alone can [27, 28]. By working together, they can draw on the broad range of resources and expertise provided by the other organisations in the network to improve community members’ health and well-being [19, 29].

Since 1986, WHO has advocated the application of these action principles as they lead to better health [30]. Most evaluation studies, however, focus on measuring outcomes, and less is known about how these action principles trigger specific outcomes in interaction with the context [31, 32]. To gain insight into the workability of these principles, an evaluation approach that is sensitive to the operational conditions of the programme as part of a larger complex system is required. This means that the evaluation should generate knowledge about what works for whom in what circumstances; this is different from the usual evaluation methods that focus on whether or not the programme has succeeded against the criteria set at the start [33, 34]. Unravelling the mechanisms underlying these action principles will expand the knowledge about community-based approaches in practice and thereby contribute to finding ways to reduce health inequalities. The aim of this evaluation study is to unravel mechanisms underlying the action principles and find combinations of contextual factors and mechanisms that trigger outcomes of interest in a CBHPP.

## Methods

### Study setting

The setting of this study is the community health promotion programme Voorstad on the Move (VoM). This local programme was one of 46 small-scale projects under the umbrella of the Healthy Futures Nearby (HFN) programme funded by the private funding organisation, FNO, with the aim of reducing health inequalities within the Netherlands [35]. From September 2016 to the end of 2019, VoM was developed and implemented in a socioeconomically deprived city district of 10,750 inhabitants in a city in the eastern part of the Netherlands. In this city district, both the SES and the health status of inhabitants are relatively low compared with other parts of the city [36]. The VoM programme pursued multiple goals. On the one hand, it aimed to improve Voorstad inhabitants’ perceived health and achieve changes in the social and physical environment in order to support healthy behaviours. At the same time, it focused on finding keys to reducing health inequalities [37]. VoM is a local programme developed and implemented with the active involvement of low SES citizens, who – in the view of health professionals – are usually hard to reach and not very interested in health promotion activities. The programme was innovative, as it differed from usual health promotion programmes in which health and lifestyle themes and activities are set by professionals. Instead, this programme shifted from being a predetermined health promotion programme with a set of health behaviour interventions to being an open approach with a focus on the action principles, citizen participation and intersectoral collaboration. The VoM programme was guided by participatory action research (PAR) [13, 37]. During the programme, stakeholders were continually involved, and different perspectives of citizens and professionals were captured and used to optimize consequent actions. As such, the development of capacities, learning, and empowerment was facilitated [38, 39].

In September 2016, the VoM programme started as a collaboration of five organisations, all part of the existing social infrastructure: the municipal health service, the Voorstad social support team (SST), the welfare organisation, the neighbourhood viability coalition, and the local sports service organisation. The programme’s driving and leading force was the VoM coalition, with representatives of the five organisations, all community workers, along with a health broker, who was an inhabitant of Voorstad, working in a self-employed capacity. The benefits of a broker role, especially in health promotion, lie in connecting stakeholders from health and non-health sectors with citizens, and subsequently stimulating an integrated community approach to address health inequalities [40]. The coalition members built a communitywide network of organisations, workers, and citizens based on the existing social infrastructure and the contacts that each of them brought in. The health broker role was essential in connecting the VoM coalition with the broader network and in facilitating citizen participation [38]. Citizens’ perspectives on health were explored with existing community groups – consisting of Voorstad inhabitants (e.g., a walking, a yoga, and a knitting group) and volunteers at a community centre and a play garden – in two group sessions with each group [39]. These community groups were actively involved in the VoM programme from the start.

**Fig. 1:**
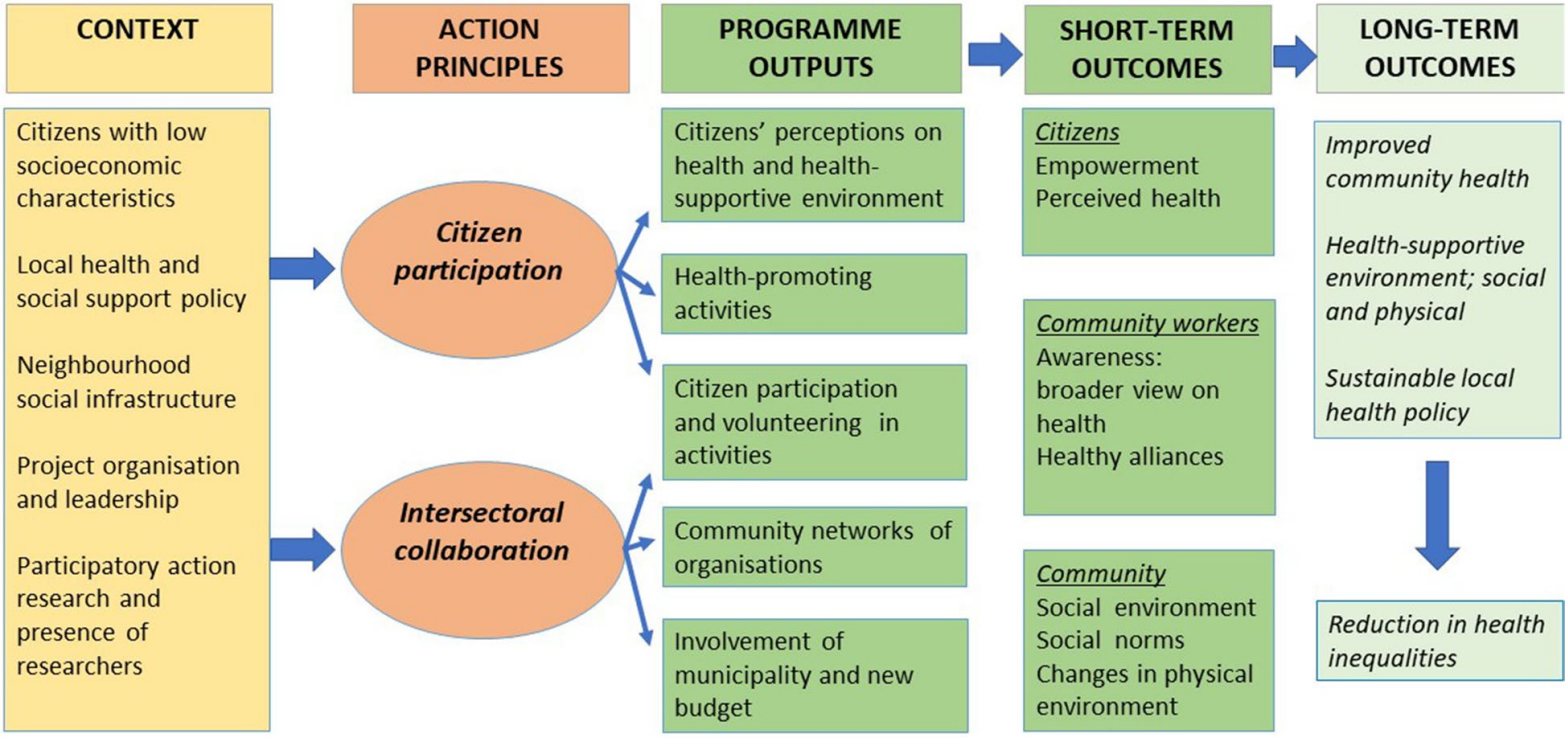
Logic model ‘Voorstad on the Move’ based on [37]

Based on the literature on community-based approaches and evaluation studies of complex community health promotion programmes [41–44], we composed an initial programme theory, represented by the a logic model (Fig. 1). This model was used for the development, implementation, and evaluation of the VoM programme [37].

The assumption is that developing and implementing health activities at different levels – i.e., citizens, community workers, and community – will result in the long term in improved perceived health, a health-supportive environment, and sustainable local health policy, and ultimately lead to a reduction in health inequalities. These long-term expected outcomes will be preceded by short-term outcomes, defined as outcomes of interest such as health literacy, healthy alliances, and changes in the physical environment. Besides intended short-term and long-term outcomes, there might be unplanned outcomes that sometimes have a greater influence on the health determinants for a community than the more narrowly focused outcome goals of projects [45]. Programme outputs that precede and generate short-term outcomes include, for example, insight into citizens’ perception on health, new health-promoting activities, an extended community network, new coalitions of primary care professionals and social support workers, and municipal involvement. Another assumption is that, by applying the citizen participation and intersectoral collaboration action principles, programme outputs will be generated, leading to short- and long-term outcomes as already explained.

#### Study design

A critical realist perspective was followed to study the citizen participation and intersectoral collaboration action principles in depth, in the single case of VoM. The critical realist perspective was used as the VoM programme was part of an open system which has implications for the outcomes being produced [46]. This method is based on the identification of outcome patterns, mechanisms, and contextual conditions that help to assess not only what works, but also for whom and in what circumstances [47–49]. The final research product from the critical realist methodology is not a statement of effect size, but rather a refinement of the programme theory [33] based on the gathered insights regarding the mechanisms that have triggered the outcomes in this specific community health promotion programme [50]. The step wise (secondary) analysis of data from multiple sources resulted in context-mechanism-outcome (CMO) configurations, related to the action principles part of the VoM programme theory [51].

### Data collection

As part of the PAR that accompanied the VoM programme, a range of qualitative data was collected throughout the programme between 2017 and 2019. In addition, data collected by researchers from the overall evaluation of the FNO HFN programme were analysed [52]. Data used in this study originated from multiple sources and methods to ensure validity. Sources and methods include for example midterm and end evaluation sessions with coalition members; individual interviews and group sessions with community workers, the health broker, and citizens; semi-annual progress reports prepared by the programme coordinator; and minutes of the VoM coalition meetings (Table 1). In addition, an activities database, in which characteristics and reports of the activities that were part of the VoM programme were registered and monitored, was consulted. All sources were used to study both action principles, except the data from the sub study ‘Benefits of participation’ (V), which was only used for the action principle citizen participation. The photo-voice study (IV), the semi-annual reports (VI) and minutes of coalition meetings (VII) were also used to clarify the context.

**Table 1:** Data collection scheme

	**Source (material)**	**Methods**	**Dates**
I	Midterm and end evaluation intersectoral collaboration [38]	6 individual interviews with VoM coalition members 4 group sessions with VoM coalition members (*n* = 6 per session)	Jan.–June 2018 Nov. 2019
II	Midterm evaluation coalition ‘*Well-being or not to be?’*^a^ [53]	Group session with community workers (*n* = 7) Individual interview with health broker	May 2019
III	Midterm and end evaluation by researchers from the overall evaluation of the FNO HFN programme [52]	2 group sessions with VoM coalition members (*n* = 6 per session)	Feb. 2018 June 2019
IV	Small-scale photovoice study about health-supportive environment [54]	3 group sessions with citizens (*n* = 15 in total), photos taken by citizens	May–June 2018
V	Sub-study ‘Benefits of participation’ [55]	12 in-depth interviews with actively participating citizens	March–April 2019
VI	Semi-annual progress reports, part of the overall evaluation of the FNO HFN programme	7 reports by the programme coordinator	Feb. 2017–Dec. 2019
VII	Monthly meetings of the VoM coalition	Minutes of the meetings 40 reports	Feb. 2017–Nov. 2019
VIII	Activities database	Project plans, reports	Jan. 2017–Dec. 2019

^a^A temporary coalition consisting of GPs, practice nurses, social support and welfare workers, facilitated by the health broker, with the aim of strengthening the collaboration between care and welfare, focusing on health and behaviour instead of illness and care

### Data analysis

Interviews and group sessions were anonymised and transcribed ad verbatim. The analysis was stepwise, data driven, and thematic [56], using Atlas-ti 22. Coding was developed based on a realist synthesis protocol with the focus on CMO configurations, looking not only at what worked, but also for whom and why it worked [46–48]. The operationalisation of the concepts – context, mechanisms, and outcomes – is illustrated in Table 2.

**Table 2:** Operationalisation of context-mechanism-outcomes concepts in the VoM programme

Concept	Theoretical definition^a^	Operational description^b^	Thematic elaboration^c^
**Context**	Refers to the fact that a relationship between causal mechanisms and their effects depends on the specific circumstances	Something (situation or condition) that existed prior to the start of the VoM programme or something happening outside control of the programme	Historical factors Organisational factors Programme-related
**Mechanisms**	Responsible for the relationship between context and outcome; the cognitive or affective responses of participants to resources offered [57]	Activities and actions taken by actors (citizens, community workers, coalition members) in the VoM programme	*Programme-related*: actions taken by community workers, VoM coalition members *Participant-related*: actions taken by citizens
**Outcomes**	Results from different layers of reality in social explanation	Results of the VoM programme; programme outputs and short-term outcomes (Fig. 1). Intended short-term and long-term outcomes as defined in the programme theory and unplanned outputs and outcomes as perceived by stakeholders (community workers and citizens)	Results at the level of: - citizens - community workers - community

^a^ Based on Pawson et al. (2005) [53], Jagosh et al. (2015) [31], Herens et al. (2017) [21]; ^b^ Based on Calò et al. (2020) [44], Marchal (2012) [54]; ^c^ Based on Herens et al. (2017)[21]

#### Step 1

Transcripts of the evaluation sessions (Sources I–III, Table 1) were coded in terms of context conditions (C), underlying mechanisms (M) in the actual programme, and outcomes observed by respondents (O) [58].

#### Step 2

Quotes coded as context were further thematised into historical-, organisational-, and programme-related codes. Quotes coded as mechanisms were also further thematised into programme- or participant-related. Quotes coded as outcomes were classified as related to citizens, community workers, or community. Each theme was further refined into subthemes and labelled as supportive ( +) or restraining (-), thus addressing the aim of differentiating and accumulating evidence on positive and negative CMO configurations [51].

The coding procedures were conducted independently by two researchers (first and second author). Both researchers found that the same phenomenon could be coded as outcome or context, or as context and mechanism, as was also found by Herens et al. [33]. Differences in coding were discussed until consensus was reached, thereby making explicit that all coding was based on the perspective of the actual VoM programme activities and processes.

#### Step 3

Sources IV and V (Table 1) were part of sub-studies in which (IV) citizens’ perspectives on the living environment were studied [54] and (V) active citizens were asked about the benefits of participation in (health promotion) activities and volunteer work [55]. Transcripts of the interviews and group sessions were also coded in terms of contexts (C), mechanisms (M), and outcomes (O). Deductive or top-down analysis was applied by the first author, based on the mechanisms and CMO configurations resulting from Steps 1 and 2.

#### Step 4

The reports and minutes of the meetings (Sources VI and VII, Table 1) were studied to check how often and in what manner the context conditions, mechanisms, and outcomes of interest found in Steps 1 and 2 had been reported.

#### Step 5

The activities database (Source VIII, Table 1) was used to gather more in-depth information about the programme outputs, e.g., the number of participants, the involvement of community workers, duration of the activities, and so forth.

**Fig. 2:**
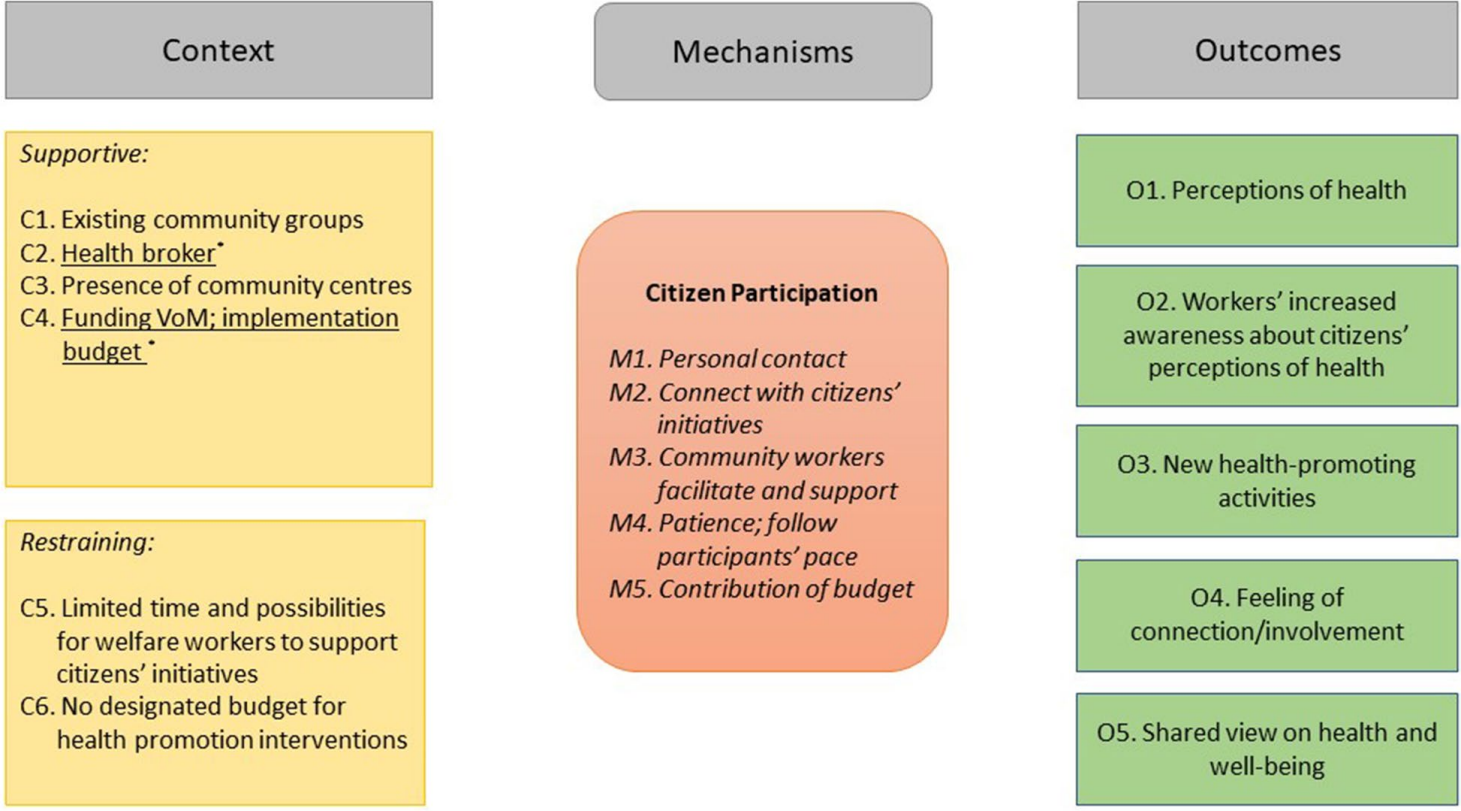
CMO configurations citizen participation

**Fig. 3:**
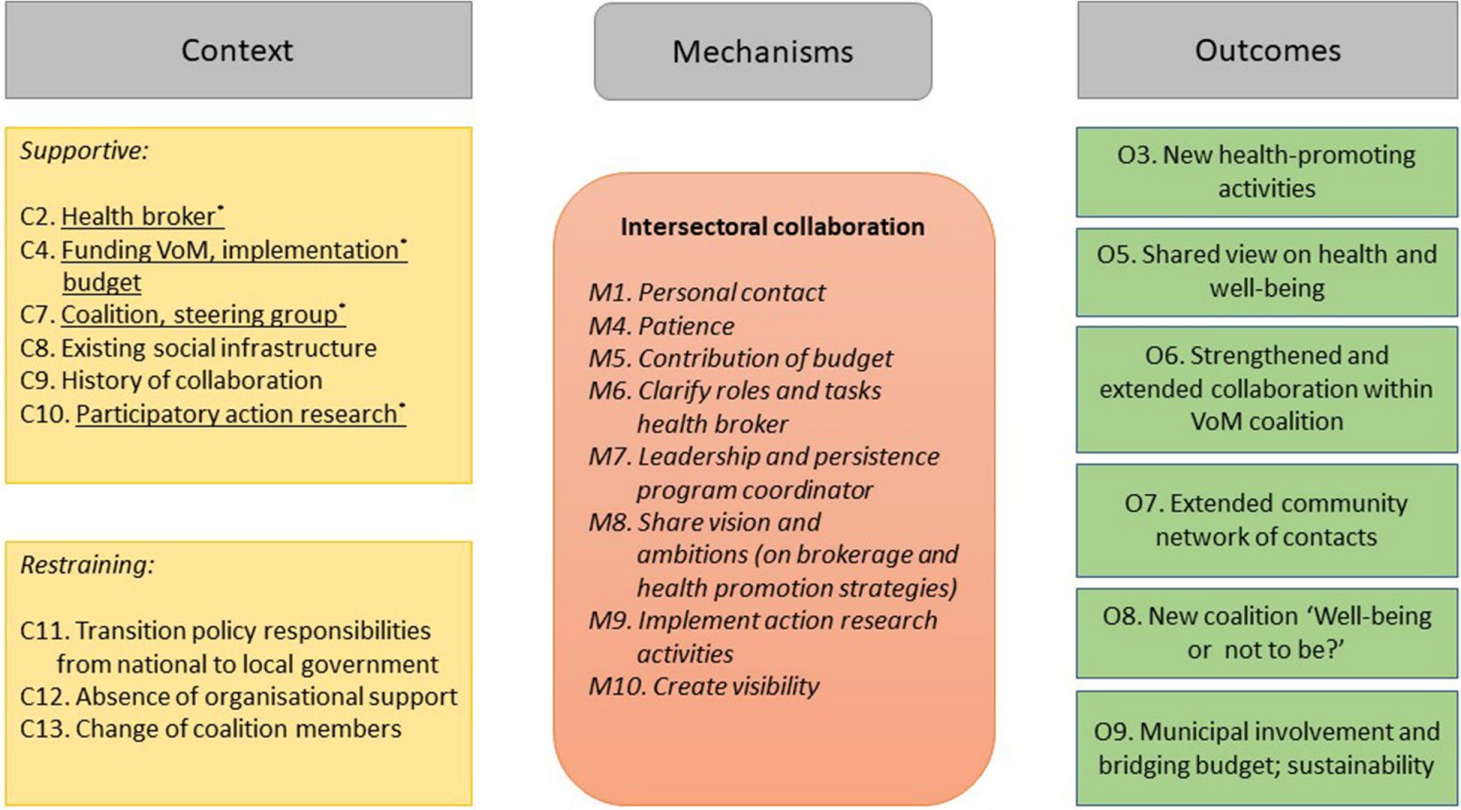
CMO configurations intersectoral collaboration

On completion of these steps, two researchers (MdJ, GW) brought together the results in an overview of the most important context conditions, mechanisms, and reported outcomes for each of the action principles. First, the citizen participation action principle was elaborated, followed by intersectoral collaboration, and this made an overlap in CMO configurations visible. The context conditions were divided into supportive and restraining, and programme-related context conditions were marked. The overviews were presented in two figures, one for each action principle (Figs. 2 and 3). Subsequently, these figures were discussed with all authors (MdJ, GW, MK, AW) and consensus was reached.

## Results

The VoM programme initiated a variety of new health-promoting activities in the city district that were organised and implemented together with the citizens, e.g., chair gymnastics, a ‘Looking for sense’ course, a toy lending point, and a reconstruction of a neighbourhood square. The activities were characterised by a great diversity in content, intensity, duration, and the number and kind of participants, e.g., the toy lending point was run by six volunteers for more than two years, five citizens were involved in the reconstruction committee for the neighbourhood square, and 40 older persons participated weekly in chair gymnastics in different groups, which continued after the VoM programme ended (see Appendix 1).

### Citizen participation

In 2017, the VoM programme started by exploring perceptions of health with existing community groups (C1), leading to insights into how citizens perceived health (O1) (Fig. 2). Social relations and interaction, physical activity, and a positive life attitude were mentioned as the most important perceptions of health.

*If you keep on moving, you experience: I feel healthy.* (Yoga group participant)

*To stay healthy, you need to think positively about all problems.* (Language group participant)

The results of these group sessions caused awareness among community workers (O2) about the significance and perceptions of health and resulted in new health-promoting activities (O3). The health broker (C2) managed to involve the community groups by utilising personal contacts (people knew her) (M1) and by taking the presence of community centres as a base (C3). New health-promoting activities (O3) resulted from these contacts and from connecting the ideas of participating citizens with existing citizens’ initiatives (M2).

The development and implementation of new health promotion activities (O3) were also facilitated and supported by community workers (M3) and often took place in the community centres located in the city district (C3). These community workers, including the health broker, built the collaboration with citizens on personal contact (M1).

*Most important is building trust. Take your time to get to know people and show your face from time to time.* (Health broker)

Citizens who participated or volunteered in activities felt connected and involved as a result (O4).

*At the course [‘Looking for sense’], I am included in the group*. (Participant).

*Knitting connects us*. (Knitting group participant).

### Intersectoral collaboration

In the first 1.5 years, the health broker was responsible for the achievement of most of the activities, always in cooperation with citizens and other community workers. Because of changes in local health and social support policies and organisational choices resulting from that, community workers found that they had limited possibilities to support citizens’ initiatives (C5). Cutbacks on welfare work and policy prioritising individual support measures meant that no designated budget for health promotion activities was available (C6). The activity budget provided by the externally funded VoM programme (M5), together with community workers’ motivation to facilitate and support (M3), helped to

*The organisation of the social support teams is turned upside down, which means that our role is unclear, which makes collaboration difficult.* (Social support team member)

*Within the existing procedures and regulations, group activities are very difficult to organise.* (Social support team member)

Later, community workers, especially the SST members, became more and more involved in the programme, eventually taking over the health broker role (C2). They anticipated the termination of the contribution of the health broker, who was temporarily subsidised as part of the VoM programme. By taking over this role, the SST members, financed by the local government to execute the social support law, were able to foster the sustainability of the activities. Several years of working together closely with Voorstad citizens, and listening to the citizens’ perspectives about health, resulted in a shared view on health and well-being (O5).

*… that health is so much more than healthy eating and physical activity, or stop smoking, but that it is mainly in the social environment and interactions.* (Health broker)

According to the coalition members, one of the most important outcomes was the strengthened and extended collaboration within the VoM coalition (O6) (Fig. 3) and with a variety of stakeholders, citizens as well as workers, in the community. Important outcome-generating mechanisms identified by the coalition members were clarity about roles and tasks within the VoM coalition (M6), the programme coordinator’s leadership and persistence (M7), and sharing vision and ambitions regarding brokerage and health promotion strategies (M8) (Fig. 3).

*I am proud of the obvious collaboration. We contact one another more easily; that’s how we do it here. We know how to find one another and that yields a lot for the neighbourhood.* (Social services team worker)

The combination of supportive context conditions like a steering group (the VoM coalition) (C7), the existing social infrastructure (C8), and a history of collaboration (C9) together with personal contact (M1) and patience (M4) resulted in the involvement of new community groups, organisations, and individuals in the coalition’s network (O7). In addition, a new coalition, named ‘Well-being or not to be?’ was formed (O8).

A restraining context condition was the unstable (policy) context in which the VoM programme was implemented because of transitions of policy responsibilities from the national to the local government (C11). Partly because of this, coalition members were confronted with cutbacks and uncertainty at the start of the VoM programme, because of the limited support from their organisations (C12) and changes in their own organisation and coalition members (C13) that diverted attention from collaboration. The implementation of action research activities (M9) helped the coalition members to recognise the processes that evolved within the coalition and made it possible to act upon them.

*I put lots of time and energy into it and then sometimes you ask yourself: does anything come out of it? The research provides the insight that it really does!* (Coalition member)

In the final year of the VoM programme, no new activities were initiated; instead, the focus of the VoM coalition and community workers was on the continuation of health promotion activities, participation by citizens and community groups, and sustaining the collaboration and the broader network. The PAR (C10) that accompanied the VoM programme increased awareness of the coalition’s achievements, like for example the organisation of the training programme ‘Leader recreation and physical activities’ that eight citizens completed successfully. Subsequently, coalition members paid more attention to creating visibility (M10) for their achievements. Visible achievements, among other things, presented in a ‘Keep Voorstad moving’ movie, contributed to the involvement of the municipality in the VoM programme and the allocation of a bridging budget (O9) by the end of the programme term, when the funding budget ended (C4). This was promising for sustaining the VoM coalition and communitywide collaboration into 2020 and beyond. With the bridging budget, two successful health promotion activities – the ‘Looking for sense’ course and chair gymnastics – could be continued in 2020.

## Discussion

In this study, we adopted a critical realist perspective to unravel mechanisms underlying the citizen participation and intersectoral collaboration action principles applied in the CBHPP, Voorstad on the Move. Because most evaluation studies still focus on measuring outcomes, this studies added value is more tangible substance to these action principles. Using a critical realist perspective helped to gain insight into what worked under the given circumstances and at the same time to identify a wide range of outcomes, as perceived by the programme’s stakeholders. The findings contributed to amplifying and enriching the initial programme theory with the most important working mechanisms in practice.

In the VoM programme and in this evaluation study, we have made the citizen participation and intersectoral collaboration action principles central. In fact, these action principles became an aim in themselves from the beginning, and therefore we managed to devote time and attention to them in practice and research. Personal contact, knowing one another, and following participants’ pace (patience) are important mechanisms underlying citizen participation and are also necessary mechanisms to build relationships and strengthen collaboration within a coalition and a community network. This indicates that these action principles overlap and cannot be told apart from the outcomes: both play a pivotal role in realising outcomes of interest. As putting the action principles into practice resulted in a range of interesting outcomes, we argue for the explicit inclusion of citizen participation and intersectoral collaboration as targets in CBHPPs [20]. Our results are in line with the observation of Lacouture et al. [50] who state that mechanisms described by users of the realist approach for evaluating interventions in public health are mainly linked to participation, collaboration, partnerships, or management processes.

The outcomes of the principle-based VoM programme triggered by the combination of context and mechanisms did not occur in a linear process. Context conditions changed constantly because of the implementation and development of the VoM programme and the mechanisms at work. Extra manpower brought in by the health broker and the programme coordinator, together with the implementation budget, were added to the existing historical and organisational context. At the same time, the VoM programme put mechanisms to work that generated a continuous interaction with outcomes, such as new health promotion activities, which in turn led to changes in the context.

This made it sometimes difficult to distinguish context conditions from mechanisms, e.g., the presence and efforts of the health broker is a context condition and the characteristics of the broker’s methods, such as personal contact and following the participant’s pace, are mechanisms. Other studies have also found that supportive contexts set in motion mechanisms that generate programme outcomes and successes, which in turn influence the contextual conditions [33, 47].

Other studies that examined the benefits and outcomes of citizen participation have found that successful and sustainable community involvement does not occur in a linear way and is challenging [59]. Citizen participation consists of complex processes influenced by a range of social and cultural factors, part of the historical and organisational context, thereby confirming once again the importance of patience and perseverance [60–62].

Looking at the mechanisms underlying the action principles reveals that health promotion professionals and other community workers need specific skills and competences to put action principles into practice, especially leadership and brokerage. Other studies on the broker role contend that skills relate to crossing sectoral borders, agenda setting, facilitating citizen participation, and entrepreneurship [40, 63, 64]. Showing leadership requires competences such as vision, setting reachable goals, being motivational and inspirational and a team player. In coalitions, the necessary skills and competences can be allocated to the members and do not have to be put on the shoulders of the health broker or the programme coordinator alone.

PAR was one of the context conditions, and implementing the research activities helped those involved in the VoM programme to gain insights into evolving processes and to make the achievements of the programme visible. Thanks to the PAR activities, along the way, awareness of outcomes grew, and subsequently confidence in the programme approach and the collaboration that had been built up increased. It helped stakeholders to recognise the processes that evolved and to act upon them and to pay more attention to the visibility of their achievements, such as the new health promotion activities and the extended and strengthened communitywide collaboration (38). Therefore, it is recommended to apply PAR as an indispensable part of a principle-based CBHPP, because it adapts to the particular situation in practice and always takes into account the perspectives of the persons involved.

### Methodological considerations

Our study indicates that using a critical realist perspective delivers an in-depth analysis of the CMO configurations and contributes to a better understanding of the workability of the citizen participation and intersectoral collaboration action principles. The insights into the mechanisms in this study concern just one case, which is both a strength and a limitation [65]. On the one hand, it has created a thorough understanding of the CMO configurations in a real-life setting, and provided a way to understand the nature of the relationship between the VoM programme and the context in which the programme is situated [46]. On the other hand, it may be hard to generalise the findings, because every CBHPP has its own characteristics and is implemented in a different context. In order to gain broader insights into the mechanisms that underly these two action principles and to make adjustments to the (initial) programme theory, more practice-based studies are needed.

The focus in this study was on two action principles mentioned in the Ottawa Charter of Health Promotion [30]. Our results indicate that citizen participation and intersectoral collaboration are crucial action principles in CBHPPs and reveal outcomes that relate to the other action principles. For example, new health promotion activities and sustainability of the programme through the municipal involvement shape healthy public policy and action. In addition, the skills and competences stakeholders require for collaboration and citizen participation need to be developed, as made explicit in one of the other Ottawa Charter principles.

One of the limitations of this study was the selection of stakeholders represented in the research. Community workers, especially the VoM coalition members, were actively involved in focus group sessions, interviews, and coalition meetings and thereby contributed to a large extent to the research. Recruitment of citizens was challenging, and this may have created bias in the total group of citizens involved in favour of those most involved in, and enthusiastic about, the VoM programme. Compared to the community workers, citizens were less represented in the study. In addition, the analytical processes used to develop the CMO configurations, although gone through with different researchers, were inherently vulnerable to subjectivity. This may have resulted in misattributions about the importance of mechanisms and the CMO configurations applied.

## Conclusions

The most notable mechanisms underlying the citizen participation and intersectoral collaboration action principles found in this study were personal contact, patience, perseverance and visibility. The PAR activities that accompanied the VoM programme were both a mechanism and a context condition and triggered outcomes of interest by helping all those involved to recognise processes, take them further, and make the outcomes visible. Applying a critical realist perspective contributed to deepening the understanding of what worked under the given circumstances and helped to identify a wide range of outcomes. Adding the underlying mechanisms to the action principles enriches the initial programme theory with insights in what works and why it works and can be helpful for health promotion professionals working in CBHPPs.

As the principle-based CBHPP added value to the existing context and brought in most of the mechanisms that triggered outcomes of interest, it is recommended to include citizen participation and intersectoral collaboration not only as action principles but explicitly as targets in a CBHPP.

## Supplementary Information

**Additional file 1.**

## Data Availability

Not applicable.

## Abbreviations

CBHPP: Community-based health promotion programme
CMO: Context-mechanism-outcome
HFN: Healthy Future Nearby (programme)
PAR: Participatory action research
SES: Socioeconomic status
SST: Social support team
VoM: Voorstad on the Move
