# Supplementary material for: Unravelling mechanisms underlying the action principles of a community-based health promotion programme: a realist evaluation
Source: Arch Public Health. 2023 Jan 19;81:9. doi: 10.1186/s13690-023-01027-0 (PMC9850519; doi:10.1186/s13690-023-01027-0)
Supplement: Supplementary file 1 — Additional file 1. [file 13690_2023_1027_MOESM1_ESM.pdf]

Appendix 1. Programme Outputs

2016	2017		2018		2019	Responsible worker*	Inhabitants involved**
Toy lending point						HB	6 V
Budget cooking workshops						HB	26 P
Mothers 'on the move'						SST	35 P
	Mancave cooking workshops					HB	23 P
	Voorstad kids					HB	65 P
	Gardening project					WF	6 P
	Outdoor playing 'Molentuin'					HB-S	15 P, 4 V
		Physical activity workshops				HB-S	15 P
		Group sessions 'Perspectives on health'				HB, HB-S, SST, AR	89 P
			Neighbourhood communication boards			HB, SST	8V
			Intersectoral collaboration and network analysis			AR	6 coalition members
			Looking for sense course			HB, SST	24 P, 1 V
			Beestenmarkt reconstruction, watertap and opening 'party'			ST, WF, HB-S	5 V
			First aid 'kids' course			WF	6 V
			Chair gymnastics			SST, HB-S	40 P
			Healthy eating workshop kids			SST, HB	8 P
			Healthy 'high tea for elderly			SST	8P
			Photovoice project: health supporting environment			HB, AR	16 P
			Benefits of Participation study			HB, AR	100 + 12 P
			Project *Well-being or not being ill?* Primary care-welfare alliance				15 CW
				Course 'healthy nutrition'		HB-S, WF	10 P

				Training: 'Leader recreation and physical activities'			7 V
					Group sessions 'How about health?"	SST, AR	29 P
					Movie 'Keep Voorstad moving'		
					Municipal involvement and bridging budget 2020		

* HB=Health broker, HB-S=Health Brokers-sports, SST=Social support team, WF=welfare worker, AR=Action Researcher

** V= volunteers, P=participants, CW = community workers

= health promoting activities

= research activities

= additional activities

= continuation activities
